# Stair Descending Exercise Using a Novel Automatic Escalator: Effects on Muscle Performance and Health-Related Parameters

**DOI:** 10.1371/journal.pone.0056218

**Published:** 2013-02-21

**Authors:** Vassilis Paschalis, Anastasios A. Theodorou, George Panayiotou, Antonios Kyparos, Dimitrios Patikas, Gerasimos V. Grivas, Michalis G. Nikolaidis, Ioannis S. Vrabas

**Affiliations:** 1 Department of Physical Education and Sport Science, University of Thessaly, Trikala, Greece; 2 Institute of Human Performance and Rehabilitation, Center for Research and Technology – Thessaly, Trikala, Greece; 3 Laboratory of Exercise, Health and Human Performance, Research Center European University of Cyprus, Nicosia, Cyprus; 4 Exercise Physiology and Biochemistry Laboratory, Department of Physical Education and Sport Sciences at Serres, Aristotle University of Thessaloniki, Serres, Greece; University of Sao Paulo, Brazil

## Abstract

A novel automatic escalator was designed, constructed and used in the present investigation. The aim of the present investigation was to compare the effect of two repeated sessions of stair descending versus stair ascending exercise on muscle performance and health-related parameters in young healthy men. Twenty males participated and were randomly divided into two equal-sized groups: a stair descending group (muscle-damaging group) and a stair ascending group (non-muscle-damaging group). Each group performed two sessions of stair descending or stair ascending exercise on the automatic escalator while a three week period was elapsed between the two exercise sessions. Indices of muscle function, insulin sensitivity, blood lipid profile and redox status were assessed before and immediately after, as well as at day 2 and day 4 after both exercise sessions. It was found that the first bout of stair descending exercise caused muscle damage, induced insulin resistance and oxidative stress as well as affected positively blood lipid profile. However, after the second bout of stair descending exercise the alterations in all parameters were diminished or abolished. On the other hand, the stair ascending exercise induced only minor effects on muscle function and health-related parameters after both exercise bouts. The results of the present investigation indicate that stair descending exercise seems to be a promising way of exercise that can provoke positive effects on blood lipid profile and antioxidant status.

## Introduction

Eccentric muscle action occurs when the muscle unsuccessfully resists elongation, acting as a brake (e.g., when the muscle lengthens to lower a load), thus, it is apparent that most daily life activities contain eccentric muscle actions. However, eccentric actions lead to physiological muscle damage, a non-permanent condition that typically begins approximately 6 hours after unaccustomed exercise, peak at 1−3 days and subside 4 to 7 days after exercise [1]. However, skeletal muscle has the ability to adapt rapidly in eccentric exercise and thus muscle damage is substantially reduced when the same type of exercise is performed several weeks later [1], a phenomenon known as the “repeated bout effect” [2].

Recently, an eccentric exercise model has been suggested by our group as a form of physical activity that may improve muscle performance and induce several health-promoting adaptations [3], [4], [5], [6], [7]. Indeed, acute eccentric exercise induced favourable changes in blood lipid profile [3] probably due to the replenishment of muscle phospholipid and triacylglycerol (TG) stores with fatty acids for the regeneration of damaged muscle fibers [8], [9], [10]. Moreover, the higher levels of resting energy expenditure [5], [11] and fat oxidation [5] found after muscle damaging exercise could be attributed to the decreased levels of serum TG. Exercise induced muscle damage also was found to decrease insulin sensitivity, which was hypothesized to be mediated by tumor necrosis factor alpha, released from inflammatory cells as a result of the disruptions to cellular integrity [12], [13]. Additionally, investigations from our group demonstrated that acute eccentric exercise may alter redox homeostasis [14], [15] due to the fact that muscle damage triggers phagocyte infiltration into muscle and generation of free radicals [16], [17], [18]. On the other hand, chronic eccentric exercise has been found to increase muscle performance [6], [7] and improve insulin sensitivity [6] probably because of the absence of muscle damage due to the adaptations taking place in skeletal muscle after repeated bouts of eccentric exercise [6], [15].

In the aforementioned studies, eccentric exercise induced by using an isokinetic dynamometer. However, the exercise performed on the isokinetic dynamometer had a number of limitations: i) the eccentric actions were performed isokinetically whereas most physiological body movements involve isotonic muscle contractions, ii) the eccentric actions were applied on isolated muscle groups whereas real life activities mobilize several muscle groups, iii) the intensity of eccentric actions were maximal instead of the moderate intensity required for most daily activities, iv) the eccentric actions induced severe muscle damage accompanied by pain and reduced functional ability much higher than those induced by most types of physical activity, v) the eccentric actions were performed by extending the knee against the force arm of the dynamometer, a movement that does not mimic adequately normal human movements, vi) the isokinetic dynamometer is a specialized device that is only available in universities and rehabilitation centers.

**Figure 1:**
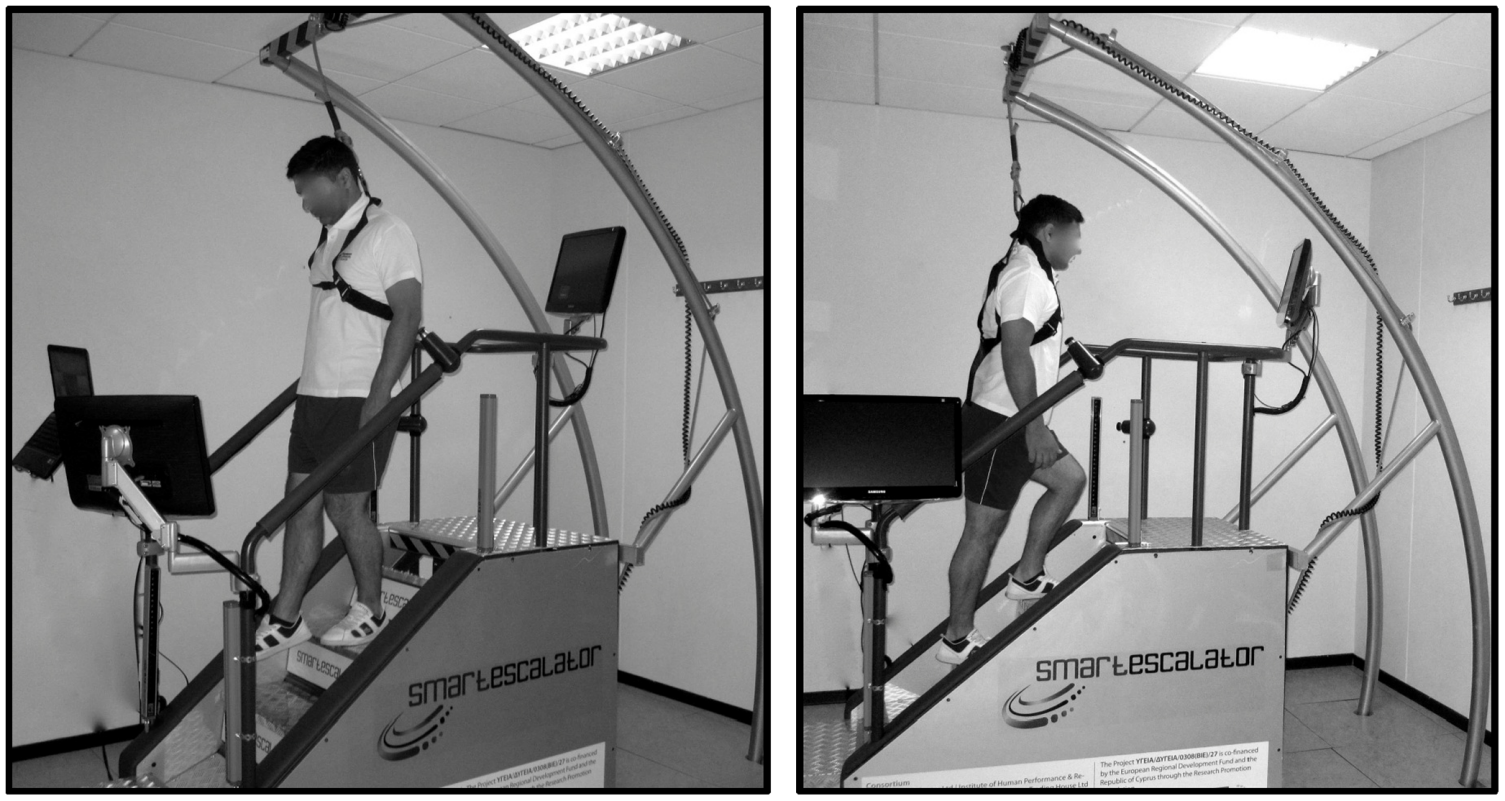
The automatic escalator device was invented, designed and constructed by our group and it is the first of its kind offering both stair descending (eccentric-biased) and stair ascending (concentric-biased) exercise.

Considering these limitations, and in order to investigate whether the benefits of pure eccentric exercise can be transferred to daily activities, a new and friendlier way to perform eccentric exercise had to be invented. To this end, we have proceeded to the design, development and construction of an automatic escalator, offering both stair descending (eccentric-biased) and stair ascending (concentric-biased) exercise (Fig. 1). Therefore, the aim of this study was to compare the effect of two repeated sessions of stair descending versus stair ascending exercise on muscle performance, insulin sensitivity, blood lipid profile and redox status in young healthy men.

## Methods

### Ethics Statement

A written informed consent to participate in the study was provided by all participants after the volunteers were informed of all risks, discomforts and benefits involved in the study. The procedures were in accordance with the Helsinki declaration of 1975, as revised in 2000, and approval was received from the Research Ethics Committee of the European University Cyprus.

### Subjects

Twenty healthy males participated in this study and were randomly divided into two equal-sized groups: a stair descending group (muscle-damaging group; n = 10) and a stair ascending group (non-muscle-damaging group; n = 10) (Table 1). The subjects participated in low intensity leisure activities (such as jogging, swimming and dancing) two to three times per week for less than three hours per week. Subjects had no experience with muscle damaging exercise for at least 6 months before the study and were not taking any medications, oral contraceptives or dietary supplements during the study period and one month before the initiation of the experiment. They were instructed to abstain from strenuous exercise for 7 days before exercise, during data collection and the interim period between the two exercise sessions. Finally, all volunteers were asked not to modify their usual way of life (including physical activity) in any aspect during the study period.

**Table 1:** Body composition and analysis of daily energy intake of stairs descending and ascending group in the first and in the second bout of exercise (mean ± SEM).

	Descending group		Ascending group	
	Bout 1	Bout 2	Bout 1	Bout 2
Age (years)	27.3±1.3		25.6±1.3	
Height (cm)	176±2.0		178±1.7	
Body mass (kg)	74.5±1.3	75.6±1.4	77.3±1.5	77.5±1.4
Body fat (%)	13.2±2.2	13.6±2.2	13.6±2.1	13.4±2.0
Fat-free mass (kg)	64.3±0.9	65.3±2.1	66.6±1.4	67.1±1.9
Energy (kcal)	2525±78	2471±85	2683±92	2506±76
Carbohydrate (% energy)	58.1±1.4	58.4±1.3	57.7±1.8	57.2±1.4
Fat (% energy)	23.5±0.9	23.1±0.7	24.5±0.7	24.9±0.6
Protein (% energy)	18.4±1.8	18.5±1.3	17.8±1.2	17.9±1.3
Vitamin A (mg, RE)	1.06±0.12	1.08±0.11	1.08±0.13	1.10±0.11
Vitamin C (mg)	125±12	131±14	122±11	122±12
Vitamin E (mg, >-TE)	9.1±0.8	8.8±1.2	9.5±1.5	9.3±1.1
Selenium (Kg)	42.4±2.7	40.4±3.1	44.3±3.2	44.1±3.0

RE, retinol equivalents; >-TE, alpha-tocopherol equivalents.

### Study Design

Each group of volunteers performed two sessions of stair descending or stair ascending exercise on the automatic escalator. Between the two exercise sessions, a three week period was elapsed. The exercise protocols were undertaken by all participants. All physiologic and biochemical measurements were determined before, immediately after as well as at day 2 and day 4 after exercise. Creatine kinase (CK) activity was determined at the same time-points except for immediately after exercise. All measurements and blood samplings were performed after both exercise bouts between 8∶00 and 10∶00 a.m. after an overnight fast and having abstained from alcohol and caffeine for 24 h. In each of the two exercise sessions, volunteers had to accomplish five sets of 5 min of stair descending or stair ascending exercise on an automatic escalator. The speed was set at 60 stairs•min^−1^ and the total number of steps undertaken during each exercise session were 1500 steps for both groups. Stair height was 20.5 mm. A 3-min interval was incorporated between sets. Each subject was familiarised with the experimental set up at least 5 days before the experimental procedures. This familiarization procedure involved 3 min at low speed (40 stairs•min^−1^) of stair descending or stair ascending exercise on an automatic escalator.

The average of the heart rate during the last minute of the last set for each subject was recorded and used for the statistical analysis. Heart rate was monitored and recorded by telemetry (Tester S610i; Polar, Electro Oy, Finland). Similarly, at the end of exercise session, rating of perceived exertion was evaluated using the Borg’s scale from 6 (very, very light) to 20 (very, very hard) (Borg 1970). The escalator used in the study has been design, developed and built from our group after receiving a grant from the Cyprus Research Promotion Foundation. This electronic escalator offers both stair descending and stair ascending exercise. It is worth mentioning that there is no similar exercise device in the market.

### Anthropometric Measurements

During their first visit, body mass was measured to the nearest 0.5 kg (Beam Balance 710; Seca, Birmingham, UK) with subjects wearing their underclothes and barefooted. Standing height was measured to the nearest 0.5 cm (Stadiometer 208; Seca, Birmingham, UK). Percentage body fat was calculated (the Siri skinfold equation was used) from seven skinfold measures (average of two measurements of each site) using a Harpenden caliper (John Bull, England).

### Muscle Function and Performance

An isokinetic dynamometer (Cybex, Ronkonkoma, NY, USA) was used for the measurement of isometric knee extensor peak torque at 90° knee flexion. The isokinetic dynamometer was calibrated weekly according to the manufacturer’s instructions. The evaluation of isometric peak torque was undertaken from the seated position (120° hip angle) and the subjects’ position was recorded for the follow-up measurements. Their lateral femoral condyle was aligned to the axis of rotation of the dynamometer while the ankle cuff was attached proximally to the lateral malleolus. Gravitational corrections were made to account for the effect of limb weight on torque measurements. Feedback of the torque produced was automatically provided by the dynamometer. During the pre-eccentric exercise evaluation of muscle performance both legs were assessed separately and the strongest one defined as the dominant and used in the follow-up measurements. The average of the 3 best maximal voluntary contractions with the dominant leg was recorded. To ensure that the subjects provided their maximal effort, the measurements were repeated if the difference between the lower and the higher torque values exceeded 10%. There was a 2-min rest between isometric efforts. The test-retest reliability of the isometric peak torque measurement was 0.97. Prior to each exercise session, subjects performed a warm-up consisting of eight-min cycling on a Monark cycle ergometer (Vansbro, Sweden) at 70 rpm and 50 W followed by five-min of ordinary stretching exercises of the major muscle groups of the lower limbs. The assessment of pain-free range of motion (ROM) was performed manually. From the seated position on the isokinetic dynamometer, the investigator moved the calf at a very low angular velocity from knee extension (0° knee angle) to the position where the subject felt any discomfort. The angle was recorded to indicate the end of the pain-free ROM. The test-retest reliability of the ROM measurement was 0.94. Additionally, each subject assessed delayed onset muscle soreness (DOMS) during a squat movement (90° knee flexion), and perceived soreness was rated on a scale ranging from 1 (normal) to 10 (very sore). The test-retest reliability of the DOMS measurement was 0.94.

### Blood Collection and Handling

Blood was collected into EDTA-containing tubes and centrifuged immediately at 1370 g for 10 min at 4°C, and the plasma was collected. The packed erythrocytes were lysed with 1∶1 (v:v) distilled water, inverted vigorously and centrifuged at 4000 g for 15 min at 4°C. Blood samples were stored in multiple aliquots at –80°C and thawed only once before analysis. All blood samples were drawn in the morning after the subjects had fasted overnight and abstained from caffeine and alcohol for 3 days before sampling.

### Blood Chemistry

Triacylglycerols (TG) and total cholesterol (TC) were assayed by enzymic spectrophotometric methods by reagent kits from Zafiropoulos (Athens, Greece). High-density lipoprotein cholesterol (HDL) was determined the same as TC after precipitation of very low density and low density lipoproteins with a reagent from Zafiropoulos (Athens, Greece). Creatine kinase (CK), uric acid and bilirubin were assayed using a kit from Zafiropoulos (Athens, Greece). These biochemical parameters were determined in triplicate with simultaneous use of a control serum from Roche (Mannheim, Germany). Each parameter was assayed on a single day to eliminate inter-assay variability. Low-density lipoprotein cholesterol (LDL) was calculated according to the following equation: LDL = TC-HDL-(TG/5) [19]. TC/HDL (considered an atherogenic index) was also calculated. Plasma glucose was assayed by the enzymic spectrophotometric method with a reagent kit from Zafiropoulos (Athens, Greece). The Insulin was determined by enzyme immunoassay using kit from DRG (Marburg, Germany). The homeostasis model assessment (HOMA) was used as a surrogate measure of insulin resistance and was calculated as fasting insulin (µU/mL) × fasting glucose (mmoI/L)/22.5. Reduced glutathione (GSH), oxidized glutathione (GSSG), thiobarbituric acid–reactive substances (TBARS), protein carbonyls, catalase, and total antioxidant capacity (TAC) were measured as previously described [20]. Albumin was determined spectrophotometrically based on the formation of a colored complex with bromocresol green reagent. Each assay was performed in duplicate and within 4 months of the blood collection.

### Dietary Analysis

To control the effect of previous diet on the outcome measures of the study and establish that after both exercise sessions the participants had similar levels of macronutrient and micronutrient intake, they were asked to record their diet for 3 days preceding the first exercise bout and repeat this diet before the second exercise bout. A written set of guidelines for monitoring dietary consumption and a record sheet for recording food intake was provided to each subject. Diet records were analyzed using the nutritional analysis system Science Fit Diet 200A (Sciencefit, Athens, Greece).

### Statistical Analysis

The distribution of all dependent variables was examined by using the Shapiro-Wilk test and was found not to differ significantly from normality. Differences on physical characteristics between the groups at baseline were examined by using an unpaired Student’s t test. A three-factor ANOVA [group (stair descending or ascending)×bout (first or second bout)×time (before exercise, post exercise, at day 2 and day 4 after exercise)] with repeated measure on time was used to analyse muscle function, blood lipids profile, insulin sensitivity and redox status. If a significant interaction was obtained, pairwise comparisons were performed by using the Sidak test method. Data are presented as means ± SEMs. The level of significance was set at α = 0.05.

## Results

### Physical Characteristics

There were no significant differences in the physical characteristics between the subjects of the two groups (Table 1). Energy, macronutrient and antioxidant intake did not differ between the two groups (Table 1). For the descending group, the heart rate for bout 1 and bout 2 was 95±8 bpm and 101±5 bpm, respectively, and the rate of exertion on Borg’s scale were 8.5±0.5 and 8.3±0.4, respectively. For the ascending group, the heart rate for bout 1 and bout 2 was 145±6 bpm and 143±7 bpm, respectively, and the rate of exertion on Borg’s scale were 12.1±0.5 and 11.9±0.3, respectively. Heart rate and Borg’s scale results indicate the less effort that is needed during stair descending compared to stair ascending. Plasma volume did not change during the 96-h post-exercise period (data not shown).

### Muscle Function and Performance

Significant time-by-group-by-bout interaction came up only for ROM [F_(1.9,69.9)_ = 4.286, P = .018] and for DOMS [F_(2.0,71.6)_ = 6.874, P = .002) while time-by-group interaction was significant for isometric peak torque [F_(2.2,78.3)_ = 3.950, P = .020], ROM [F_(1.9,69.9)_ = 11.298, P<.001], DOMS [F_(2.0,71.6)_ = 29.031, P<.001] as well as CK [F_(1.4,51.3)_ = 8.557, P = .002]. Finally, time-by-bout interaction was significant for ROM [F_(1.9,69.9)_ = 8.557, P = .001], DOMS [F_(2.0,71.6)_ = 8.993, P<.001] as well as CK [F_(1.4,51.3)_ = 9.118, P = .001].

Significant main effect of time came up for all muscle damage and muscle function parameters [isometric torque: F_(2.2,78.3)_ = 65.164, P<.001; ROM: F_(1.9,69.9)_ = 55.967, P<.001; DOMS: F_(2.0,71.6)_ = 70.981, P<.001; CK: F_(1.4,51.3)_ = 35.097, P<.001], while significant main effect of group and bout came up for ROM [F_(1,36)_ = 16.467, P<.001 and F_(1,36)_ = 17.280 respectively], DOMS [F_(1,36)_ = 49.383, P<.001 and F_(1,36)_ = 14.429, P = .001 respectively] and CK [F_(1,36)_ = 10.556, P = .003 and F_(1,36)_ = 8.451, P = .003 respectively].

**Figure 2:**
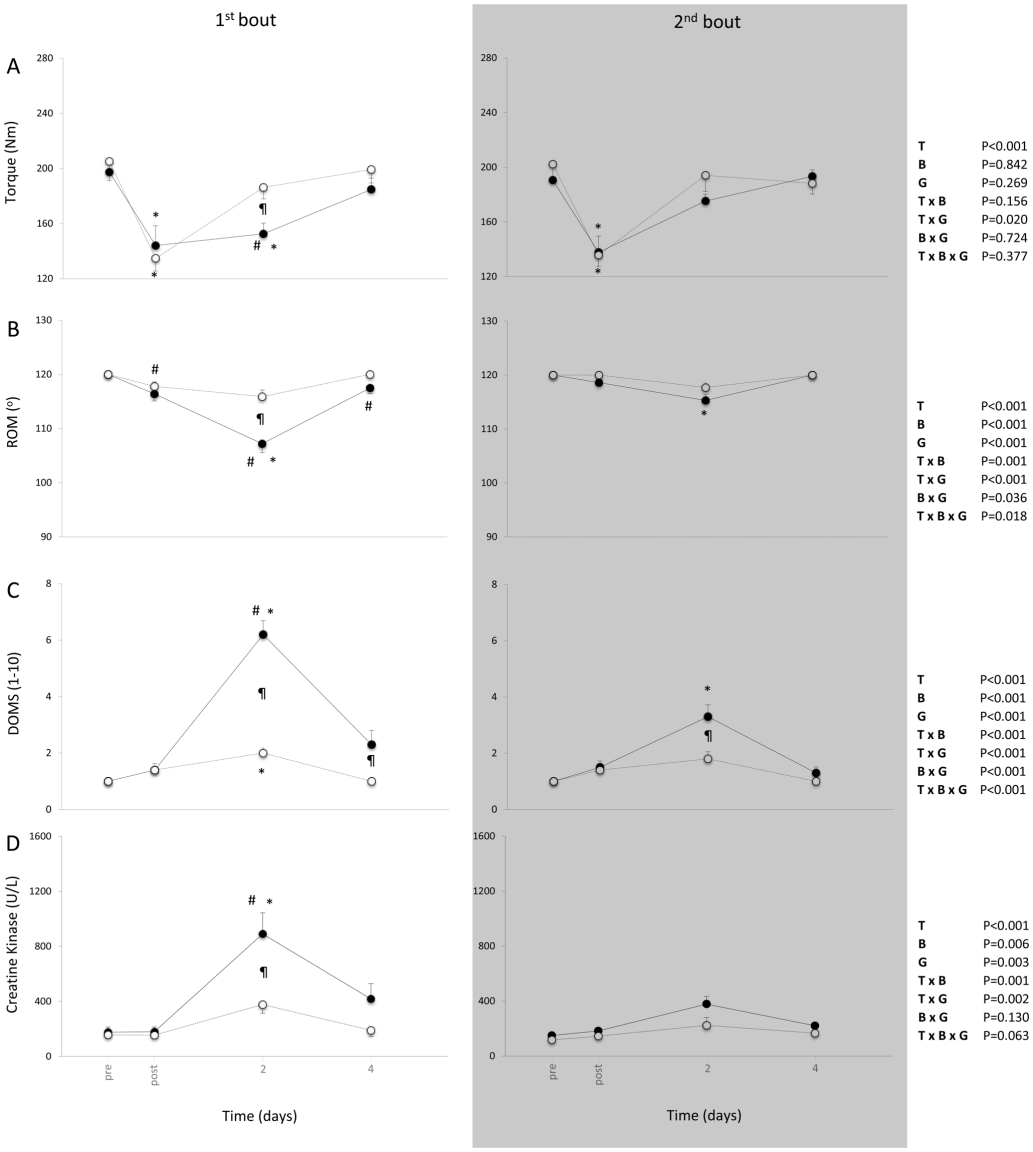
The effect of the two bouts of descending (filled circles, solid line) and ascending (open circles, dash line) exercise on torque (A), ROM (B) DOMS (C) and CK (D) (mean ± SEM). *Significantly different from the pre-exercise value in the same session (*P*<0.05). ^#^Significant difference between session 1 and session 2 at the same time-point (*P*<0.05). ^¶^Significant difference between ascending and descending group in the same session at the same time point (*P*<0.05).

In the descending group, bout 1 induced significant alterations in isometric peak torque, ROM, DOMS and CK indicating that muscle damage did occur, while these changes were diminished after bout 2 as a result of the repeated bout effect (Fig. 2). In the ascending group, bout 1 induced minor alterations in muscle damage indices while bout 2 did not affect muscle damage indices. Significant differences between the two groups were observed only at bout 1 while in the second exercise bout only DOMS was observed to be higher in the stair descending group compared to the stair ascending group.

### Insulin Sensitivity

Significant time-by-group-by-bout interaction found for insulin [F_(2.3,83.4)_ = 6.682, P = .001] and HOMA [F_(2.1,75.5)_ = 4.631, P = .012], significant time-by-group interaction found for glucose [F_(2.4,85.1)_ = 5.784, P = .003] and HOMA [F_(2.1,75.5)_ = 5.716, P = .004], while, significant time-by-bout interaction found only for glucose [F_(2.4,85.1)_ = 4.160, P = .014].

**Figure 3:**
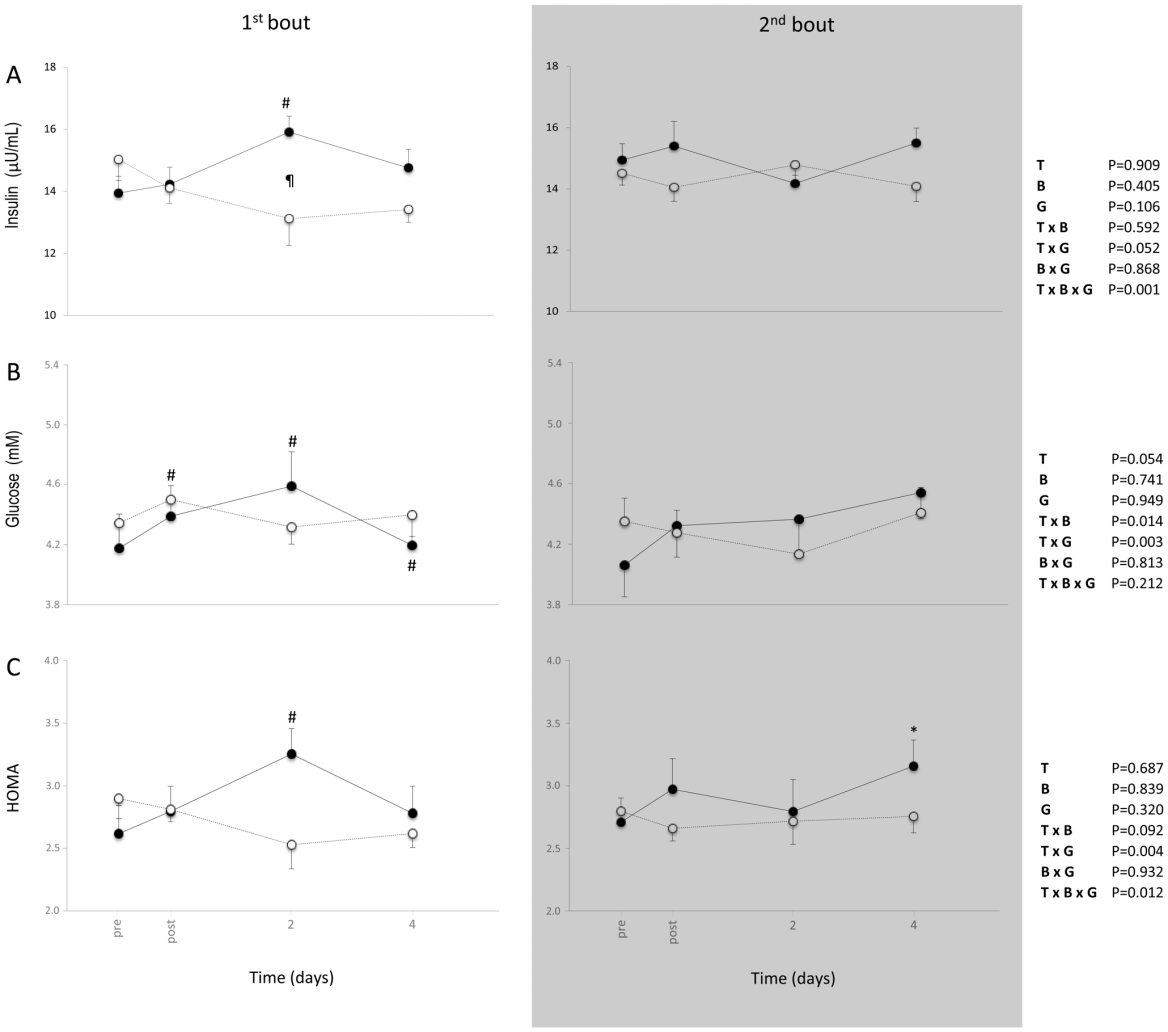
The effect of the two bouts of descending (filled circles, solid line) and ascending (open circles, dash line) exercise on Insulin (A), glucose (B) and HOMA (C) (mean ± SEM). * Significantly different from the pre-exercise value in the same session (*P*<0.05). ^#^Significant difference between session 1 and session 2 at the same time-point (*P*<0.05). ^¶^Significant difference between ascending and descending group in the same session at the same time point (*P*<0.05).

In the stair descending group, bout 1 induced significant elevations of insulin, glucose and HOMA in plasma at day 2 post exercise, indicating insulin resistance probably due to the presence of muscle damage (Fig. 3). In bout 2 the increases in insulin sensitivity indices were significantly milder compared to bout 1. Stair ascending group did not affect insulin sensitivity after bout 1 and revealed no differences between the bout 1 and bout 2 of exercise. Concluding, stair descending exercise systematically caused greater elevations in insulin sensitivity indices compared to stair ascending exercise.

### Blood Lipid Profile

Significant time-by-group-by-bout interaction appeared for LDL [F_(2.3,83.6)_ = 3.864, P = .020], TC/HDL [F_(2.7,97.9)_ = 2.944, P = .042] and for HDL [F_(2.8,100.3)_ = 4.588, P = .006] and significant time-by-group interaction appeared for TG [F_(2.8,101.8)_ = 11.649, P<.001], LDL [F_(2.3,83.6)_ = 5.208, P = .005] and TC/HDL [F_(2.7,97.9)_ = 3.015, P = .038]. Significant main effect of time found for TG [F_(2.8,101.8)_ = 2.927, P = .040] and LDL [F_(2.3,83.6)_ = 5.149, P = .005].

**Figure 4:**
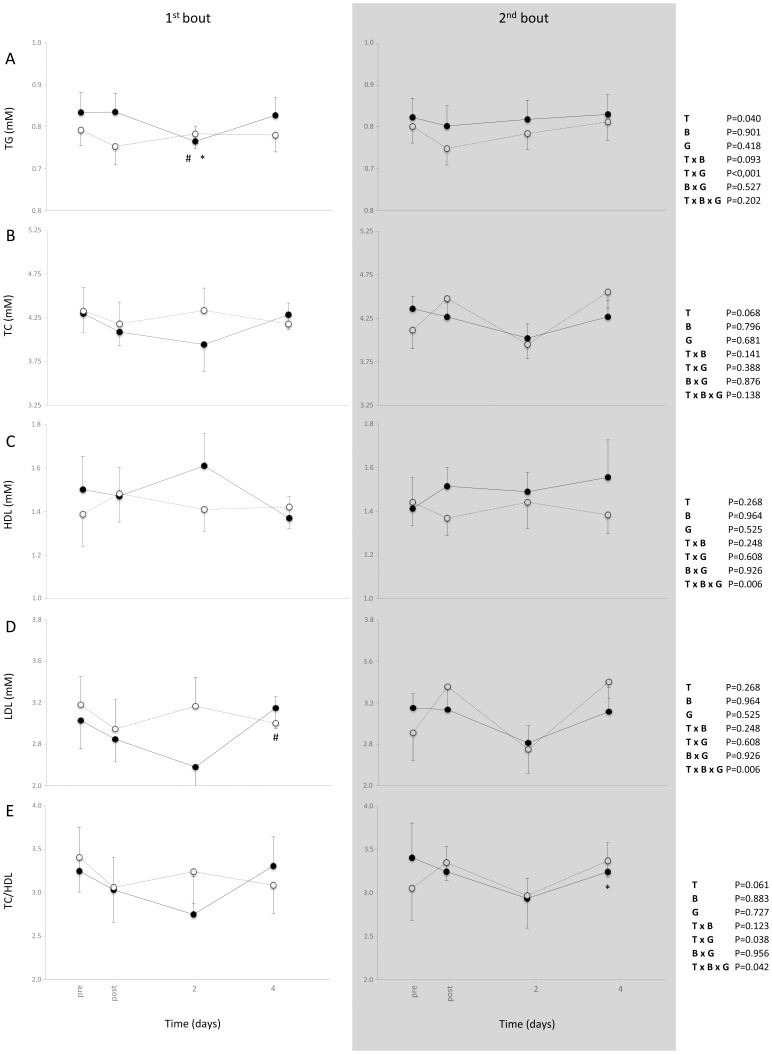
The effect of the two bouts of descending (filled circles, solid line) and ascending (open circles, dash line) exercise on TG (A), TC (B) HDL (C) and LDL (D) concentration as well as TC/HDL (E) ratio (mean ± SEM). *Significantly different from the pre-exercise value in the same session (*P*<0.05). ^#^Significant difference between session 1 and session 2 at the same time-point (*P*<0.05).

The first bout of stair descending exercise revealed positive alterations in blood lipid profile, as can be judged by the decreased values of TG, TC, LDL, TC/HDL ratio and the increased values of HDL (Fig. 4). The second bout of stair descending exercise caused less and fewer alterations in blood lipid profile compared to the first bout. The stair ascending exercise did not cause any effect on blood lipid profile after both bouts of exercise.

### Blood Redox Status

Significant time-by-group-by-bout interaction came up for GSSG [F_(2.7,96.1)_ = 3.023, P = .039], GSH/GSSG ration [F_(2.3,83.9)_ = 3.898, P = .019], TBARS [F_(2.5,89.6)_ = 7.844, P<.001] and for TAC [F_(2.5,90.0)_ = 4.727, P = .007], significant time-by-group interaction came up for protein carbonyls [F_(2.7,96.4)_ = 7.006, P<.001], TBARS [F_(2.5,89.6)_ = 6.649, P = .001], uric acid [F_(2.8,100.5)_ = 18.229, P<.001] and bilirubin [F_(2.8,100.1)_ = 4.950, P = .004] and finally, significant time-by-bout interaction came up for protein carbonyls [F_(2.7,96.4)_ = 3.336, P = .027], uric acid [F_(2.8,100.5)_ = 3.787, P = .015] and bilirubin [F_(2.8,100.1)_ = 3.437, P = .023]. Significant main effect of time came up for TBARS [F_(2.5,89.6)_ = 8.024, P<.001], TAC [F_(2.5,90.0)_ = 5.983, P = .002], uric acid [F_(2.8,100.5)_ = 9.192, P<.001] and bilirubin [F_(2.8,100.1)_ = 6.130, P = .001].

Stair descending exercise caused alterations (though mostly non-significant) in glutathione status after bout 1 as indicated by the decreased GSH, the increased GSSG and the decreased ratio of GSH/GSSG while these alterations were lower after bout 2 (Table 2). Stair ascending exercise did not induce any alterations in glutathione status. Protein carbonyls and TBARS significantly elevated in the stair descending group after bout 1, while no significant elevation was observed after bout 2. Stair ascending exercise did not modify the levels of protein carbonyls and TBARS after both bouts of exercise. The significant differences found in protein carbonyls and TBARS between the two groups indicate that descending group caused greater protein and lipid oxidation compared to the stair ascending group. Finally, stair descending exercise caused significant elevation in TAC and uric acid after bout 1, however, this elevation was milder after bout 2. Stair ascending exercise did not cause any alterations in TAC and uric acid after both exercise bouts. Neither type of stair exercise caused any significant alteration in albumin irrespective of the exercise bout while bilirubin in the bout 1 of the descending group found to be significant elevated at day 2 compared to the pre exercise values which was also significant different compared with the bout 2.

**Table 2:** Redox status indices at pre exercise, immediately post exercise and at day 2 and day 4 post exercise after the stair descending and stair ascending exercise (mean ± SEM).

	1^st^ Bout				2^nd^ Bout				Main effects and interactions						
	Pre	Post	Day 2	Day 4	Pre	Post	Day 2	Day 4	T	B	G	T×B	T×G	B×G	G×B×T
**GSH (µmol/g Hb)**															
Descending	3.00±0.27	2.92±0.25	2.85±0.27	2.99±0.26	2.89±0.28	3.08±0.25	2.64±0.26	3.13±0.27	.091	.968	.321	.244	.481	.940	.990
Ascending	2.70±0.26	2.82±0.17	2.73±0.27	2.66±0.17	2.57±0.20	2.98±0.15*	2.61±0.21	2.84±0.16							
**GSSG (µmol/g Hb)**															
Descending	0.40±0.06	0.32±0.04	0.48±0.06#	0.38±0.05	0.39±0.06	0.41±0.05	0.34±0.04	0.33±0.02	.115	.891	.100	.148	.538	.562	.069
Ascending	0.31±0.03	0.35±0.05	0.32±0.08	0.28±0.02	0.37±0.04	0.31±0.03	0.36±0.04	0.27±0.06							
**GSH/GSSG**															
Descending	9.20±1.63	10.05±1.01	6.65±0.82	9.54±1.93	8.21±1.03	8.10±0.91	8.03±0.82	9.67±0.94	.054	.590	.089	.083	.225	.842	.019
Ascending	9.84±1.39	10.28±1.93	13.19±2.22¶	10.21±1.24	7.62±1.02	10.51±1.30	8.00±1.01	14.30±2.52							
**Carbonyls (nmol/mg pr.)**															
Descending	0.48±0.08	0.52±0.08	0.76±0.09*	0.58±0.09	0.55±0.10	0.45±0.06	0.61±0.07	0.49±0.07	.125	.524	.959	.028	<.001	.834	.147
Ascending	0.52±0.09	0.63±0.07	0.50±0.06¶	0.62±0.04	0.58±0.10	0.63±0.08	0.54±0.06	0.40±0.11							
**TBARS (µM)**															
Descending	9.69±0.69#	10.29±0.65	13.33±0.97* #	10.97±0.71	8.38±0.64	10.35±0.73	9.48±0.74	9.00±0.70	<.001	.304	.416	.399	.001	.959	<.001
Ascending	11.13±0.80	12.19±0.78¶	9.98±0.88¶	10.35±0.80	9.99±0.57	10.78±0.69	11.02±0.50¶	8.72±0.90							
**Catalase (µmol/min/mg Hb)**															
Descending	150.2±9.79	154.1±10.77	168.2±8.58#	160.5±7.43	147.5±7.58	153.3±8.94	142.6±6.02	158.8±9.72	.152	.380	.589	.757	.332	.859	.001
Ascending	153.2±7.58	163.0±5.61	145.5±9.28	150.4±7.12	140.1±9.02	146.9±7.85	157.0±6.60	147.6±4.81							
**TAC (mM DPPH)**															
Descending	0.57±0.08	0.50±0.06	0.80±0.07* #	0.67±0.11	0.49±0.09	0.63±0.07	0.56±0.09	0.59±0.07	.002	.621	.421	.216	.654	.675	.007
Ascending	0.45±0.09	0.58±0.08	0.62±0.08	0.53±0.12	0.54±0.10	0.47±0.09	0.63±0.10	0.51±0.06							
**Uric acid (µM)**															
Descending	301.7±30.1	288.7±29.9	346.8±26.8*	327.2±23.5	288.9±21.1	308.4±18.9	324.8±20.3*	304.2±19.5	<.001	.704	.239	.015	<.001	.406	.114
Ascending	274.8±17.7	270.0±19.2	261.3±17.0¶	288.6±17.8	296.6±18.9	300.7±19.6	284.1±17.2	315.2±19.2							
**Albumin (µM)**															
Descending	4.25±0.22	4.37±0.23	4.20±0.26	4.43±0.21	4.42±0.22	4.49±0.32	4.34±0.32	4.64±0.24	.115	.475	.468	.398	.160	.996	.217
Ascending	4.63±0.22	4.50±0.19	4.31±0.25	4.46±0.28	4.53±0.21	4.77±0.17	4.69±0.12	4.56±0.14							
**Bilirubin (µM)**															
Descending	9.79±0.30	9.41±0.18	11.69±0.43* #	10.06±0.25	10.18±0.34	10.49±0.31	10.85±0.44	9.48±0.36	.001	.742	.754	.023	.004	.773	.235
Ascending	10.32±0.34	9.30±0.63	9.98±0.55	10.63±0.48	10.76±0.48	9.81±0.39	10.49±0.50	9.91±0.38							

GSH, reduced glutathione; GSSG, oxidized glutathione; TAC, total antioxidant capacity; TBARS, thiobarbituric acid–reactive substances. G×B×T: 3-way interaction for group, bout and time; G×B: 2-way interaction for group and bout; G×T: 2-way interaction for group and time; B×T: 2-way interaction for bout and time; G: Main effect of training group; B: Main effect of bout; T: Main effect of time.

* Significantly different from the pre-exercise value in the same bout (P<0.05).

# Significant difference between bout 1 and bout 2 at the same time-point (P<0.05).

¶ Significant difference between ascending and descending group in the same bout at the same time point (P<0.05).

## Discussion

Mobility problems in the aged may lead to falls, which in turn may result in hip fractures [21]. The majority of falls occur while negotiating stairs, especially during stair descending [22], while the United States Department of Health and Human Services suggests that physical activity can lower the risk of falls [23], [24]. One of the main mobility challenges facing older individuals are the fear of stair ascending and, particularly, stair descending negotiation. In order to help people dealing with these fears, an automatic escalator was designed, constructed and used in the present investigation. The high safety standards and the friendly use are the main advantages of the device. The novelty feature of this automatic escalator is its capability to move in both directions (i.e., stair descending and stair ascending exercise). Therefore, the main aim of the present investigation was to compare the effect of two repeated sessions of stair descending versus stair ascending exercise on muscle performance, insulin sensitivity, blood lipid profile and blood redox status in young healthy men. It was found that the first bout of stair descending exercise caused muscle damage, insulin resistance and affected positively blood lipid profile; however, after the second bout the alterations in all parameters were diminished. Regarding the stair ascending mode, it was found to have only marginal effects on muscle function and health parameters after both exercise bouts.

### Muscle Function and Performance

The first bout of stair descending exercise caused greater muscle malfunction than stair ascending exercise as can be inferred from the indirect indices of muscle damage (i.e., ROM, DOMS and CK). On the other hand, both types of stair exercise caused similar reductions in muscle performance immediately after the first bout, whereas, muscle torque was recovered faster in the ascending group. The negative effects of the first bout in muscle function and performance were subsided after the second bout, especially in the stair descending group, due to the repeated bout effect [1], [2], [3].

### Insulin Sensitivity

The main role of insulin is to lower blood glucose levels by facilitating glucose uptake mainly into skeletal muscle, liver and fat tissue. In the present investigation, the elevation of glucose, insulin and HOMA in blood after the first bout of the stair descending exercise indicates the presence of insulin resistance. High plasma levels of insulin and glucose are two of the major manifestations of metabolic syndrome [25]. It is irony that insulin resistance may occur after unaccustomed exercise in sedentary people. Fortunately, insulin resistance is a transient condition that follows unaccustomed exercise due to its damaging effect on muscle fibres [6], [13], [26]. Indeed, even after the second bout of stair descending exercise, insulin resistance was found to be much lower compared to the first bout, probably because the magnitude of muscle damage was equally lower due to the repeated bout effect [4], [6], [27]. The major limitation of the present investigation relies on the fact that the exercise was performed only twice. A chronic exercise protocol of stair ascending and stair descending exercise could have provided more evidence regarding the effects of this kind of exercise on insulin sensitivity. In fact, in a recent investigation from our group, decreased resting levels of insulin, glucose and HOMA have been reported after chronic muscle-damaging exercise [6].

### Blood Lipid Profile

Hyperlipidemia is defined as the presence of abnormal levels of lipids and/or lipoproteins in the blood [28]. Favourable changes in the concentration of blood lipids have been observed after a single aerobic exercise session [29], [30], [31], [32] as well as after unaccustomed resistance exercise [3], [33], [34]. The only significant effect on blood lipid profile appeared in TG (decreased levels) after the first bout of stair descending exercise. However, after stair descending exercise a trend for positive effects on blood lipid profile appeared only after the first bout of exercise. On the other hand, stair ascending exercise did not modify blood lipid profile after both bouts of exercise. These findings agree with those of previous works from our group, where it has been found that pure eccentric exercise induced much more beneficial effects on blood lipid profile than pure concentric exercise [3], [4], [6]. A chronic exercise intervention is imperative in order to compare the effects of stair ascending and stair descending exercise on blood lipid profile, considering that chronic aerobic exercise [29] as well as chronic resistance eccentric exercise [6] can favourably influence the level of circulating lipids.

### Blood Redox Status

Oxidative stress is defined as an increase in the level of reactive species and/or oxidant biomarkers [35]. Oxidative stress is thought to be involved in the development of many diseases or may exacerbate their symptoms like cancer [36], atherosclerosis and heart failure [37] as well as chronic fatigue syndrome [38]. On the other hand, free radicals are used by the immune system as a way to kill pathogens [39] and short-term episodes of oxidative stress may be important in delaying some aspects of aging [40]. Regarding exercise induced oxidative stress, there is increasing evidence to support the hypothesis that free radicals play an essential role in regulating, among others, hypertrophy [41], muscle force [42], muscle fatigue [43], muscle damage and repair [3], glucose uptake [44] as well as adaptations to chronic exercise [45]. In the present investigation, some significant alterations in oxidative stress appeared only after the first bout of stair descending exercise. Stair ascending exercise did not cause significant changes in redox homeostasis. However, it is clear that adaptations took place in skeletal muscle judging by the lower alterations in oxidative stress biomarkers after the second exercise bout compared to the first one. Stair descending exercise on the escalator induced qualitatively similar but quantitatively lower alterations in redox homeostasis compared to unaccustomed exercise on the isokinetic dynamometer [15], [46].

### Conclusion

Several research groups have investigated the effects of stair ascending exercise on rehabilitation from injuries and health [47], [48], [49], [50], [51]. To our knowledge, stair descending exercise has been used as a tool to assess muscle function and performance and not as a mode of exercise [52], [53], [54], [55], [56]. The results of the present investigation indicate that stair descending exercise seems as a promising mode of exercise that can provoke positive effects on blood lipid profile and antioxidant status. However, more research is needed in order to evaluate the possible health promoting effects of stair descending exercise after a chronic experimental intervention. Finally, the effects of SmartEscalator™ on human health should be compared with other traditional types of exercises (such as running on a treadmill, cycling or rowing on an ergometer).
